# Scaling up and scaling down supply chains in volatile resource-based economies

**DOI:** 10.1177/0269094221993439

**Published:** 2021-02-16

**Authors:** Laura Ryser, Sean Markey, Greg Halseth

**Affiliations:** 6727 University of Northern British Columbia, Canada; 1763 Simon Fraser University, Canada; 6727 University of Northern British Columbia, Canada

**Keywords:** business, resilience, rural, staples, supply chain

## Abstract

The growth of mobile workforces to support diversified resource extraction activities, compared to historically single-industry towns, represents a key change in rural and remote resource landscapes that has accelerated since the 1980s. Mobile workforces can present many opportunities to rural communities and economies. However, the capacity, viability and competitiveness of rural-based businesses to engage in supply chains serving mobile labour may be undermined by limited attention to how businesses manoeuvre downturns while maintaining a level of readiness to recover and scale-up in order to meet emerging mobile workforce needs. Drawing upon interviews with businesses in Fort St. John, British Columbia, Canada, our research uses the concept of resiliency to examine challenges and strategies associated with business capacity and agility to scale-up and scale-down in response to changing economic conditions associated with large-scale mobile workforces and related economic sectors. Our findings suggest that the capacity to scale-up and scale-down is shaped by capital, human resource and infrastructure strategies, inventory management and contract management strategies. Industry and state policies may also play a role supporting the conditions that will improve the agility, capacity and readiness of businesses operating in volatile resource-based economies.

## Introduction

Resource-based regions are undergoing significant restructuring as they experience more fluid flows of capital and labour (Haslam McKenzie and Rowley, 2013). Globalization, industrial restructuring, labour shortages and competitive labour markets, improvements with transportation and communication, reduced senior government roles and evolving regulations in remote resource regions have been accelerating a shift towards the use of mobile workforces to support industrial projects in resource-based regions (Rolfe and Kinnear, 2013; Tonts, 2010). Large mobile workforces present many opportunities for local businesses through the provision of transportation; security; catering; communications; and construction, operations and decommissioning of accommodations. As resource development projects are mobilized, however, industry outsourcing with established global supply chain networks may exclude local businesses and communities from the economic benefits of large-scale industrial development (Tonts et al., 2012). The capacity, viability and competitiveness of local businesses to engage in supply chains serving mobile workforces is further undermined by limited attention to how these businesses manoeuvre downturns in boom and bust cycles, while also seeking to maintain a level of capacity and readiness to quickly scale-up and meet industry needs in volatile markets. The ability of rural-based businesses to engage in this type of economic activity has significant implications for rural local economies.

The purpose of this article is to examine the challenges and strategies associated with local business capacity and agility to scale-up and scale-down in response to changing conditions associated with large-scale industrial projects and related mobile workforces. The research draws upon interviews with businesses in Fort St. John, British Columbia (BC), a resource town in the Northeast region dealing with large-scale resource projects in hydro development and the oil and gas sector. We draw upon Staples Theory and supply chain resilience literatures to inform a useful approach for examining decisions guiding supply chain strategies in volatile resource-based economies. Collectively, these literatures inform inquiries about how some businesses are better positioned to manoeuvre within cyclical economic pressures and rapidly changing industry strategies to their operations. While our research setting is specific to the sectors and contextual conditions of northern BC, it is our hope that the findings and discussion will be relevant to other industrialized rural settings.

We begin by situating our research within the volatility of staples-dependent, resource-based economies. The article then examines the literatures guiding the readiness to scale-up and engage in supply chains and the challenges to manage risks associated with supply chain disruptions. We describe a practical framework for examining agility and resiliency to assess not only the readiness of businesses to engage in supply chains, but also their capacity to respond and recover across boom and bust cycles. Our findings suggest that the capacity to scale-up and scale-down in response to changing conditions is shaped by financial capital, human resource and infrastructure strategies, inventory management and contract management strategies. Risks associated with payment structures, economic downturns, project delays, cancelled contracts, or senior government decisions about major industry projects, however, challenge the management of equipment, inventories and accommodation assets. As businesses engage in these supply chains, the recruitment and retention of skilled labour is not only challenging during periods of economic decline, but also as economic growth re-emerges and labour expectations test a recovering local business sector capacity. Moving forward, there is a need to focus on creating the conditions that will improve the agility, capacity, readiness and competitiveness of businesses operating in volatile resource-based economies.

## Staples volatility

Resource-based regions are characterized by volatility as fluctuations in commodity prices can make both ‘boom’ and ‘bust’ periods unpredictable for stakeholders (Haslam McKenzie et al., 2009; Woodworth, 2018). In seeking to better understand the volatile economic conditions under which businesses operate in rural regions, we draw upon Staples Theory to understand the dependence of rural economies on the export of raw natural resources (known as staples) that form the basic inputs for value-added manufacturing processes (Innis, 1933). In rural and remote regions, strong resource commodity prices can spur robust economic growth. Staples-based economies, however, are vulnerable to demands and prices that are set in countries equipped with more advanced technology and manufacturing infrastructure (Nelsen et al., 2010). Over time, industry consolidates its control over a stable and predictable supply of raw resources, and takes advantage of more fluid flows of capital and labour (Ryser et al., 2019). If an industry is the only employer in town, local contractors and suppliers encounter significant pressures to accept lower contract fees when industry profits are threatened by global market fluctuations (Halseth and Ryser, 2018). Local businesses have limited options as they are so intimately tied to the company for their survival. In this context, resource-dependent communities are stuck in the ‘staples trap’, bound to fluctuating, and often declining, resource revenues through the loss of jobs, taxes and related investments, and have difficulty in developing the necessary mechanisms to respond to local and regional change (Carson, 2011). In contrast, however, renewed industrial investments have spurred rapidly growing ‘boomtowns’ in resource-based regions, often characterized by employment and population growth in terms of permanent residents and ‘shadow’ populations from the influx of mobile workers (Fernando and Hearne, 2017; Woodworth, 2018).

Studies of rural and regional development focus upon the stresses associated with either economic booms or busts of a particular resource sector in lieu of how resource-based industries impact each other (Freudenburg and Frickel, 1994). Ryser et al. (2014) argue, however, that owing to more flexible modes of production, resource-based economies experience on-going and alternating up- and down-swings in economic activity across different sectors. Rather than coping, at different times, with the pressures of growth or contraction, communities must adapt to a perpetual state of readiness to react and plan for both on a much more compressed timeline through the ongoing presence of ‘regional waves’. In this context, businesses in resource-dependent regions are intimately tied to the turn-key operations of industry and fluctuations with large-scale mobile workforces used to support construction and operations as multi-national corporations are able to idle and restart their construction and production facilities quickly in response to market activity (Markey et al., 2012). Brown et al. (2005) further argue that people’s evaluation of, and responses to, change occur not only during economic up- or down-swings, but also during the ‘recovery’ cycles that occur in between these periods.

**Figure 1.**
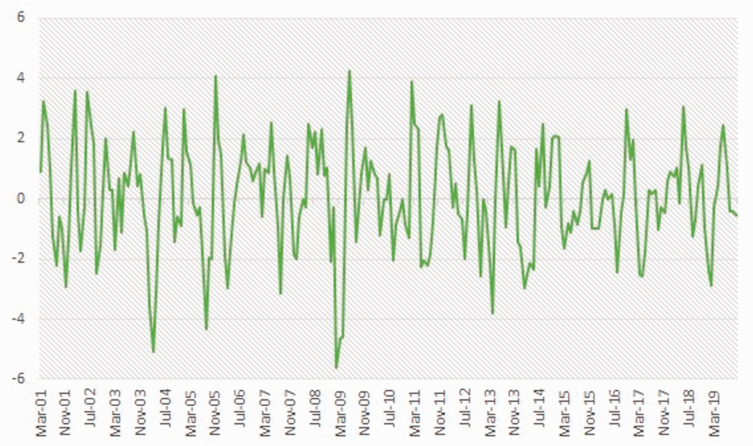
Northeast Economic Region, BC. Per cent change in employment compared to previous month (3 month moving averages; unadjusted for seasonality for last five months of data. Source: Statistics Canada. 2019. Table 14-10-0293-01: Labour force characteristics by economic region, three-month average, unadjusted for seasonality, last five months. Ottawa: Statistics Canada.

**Figure 2.**
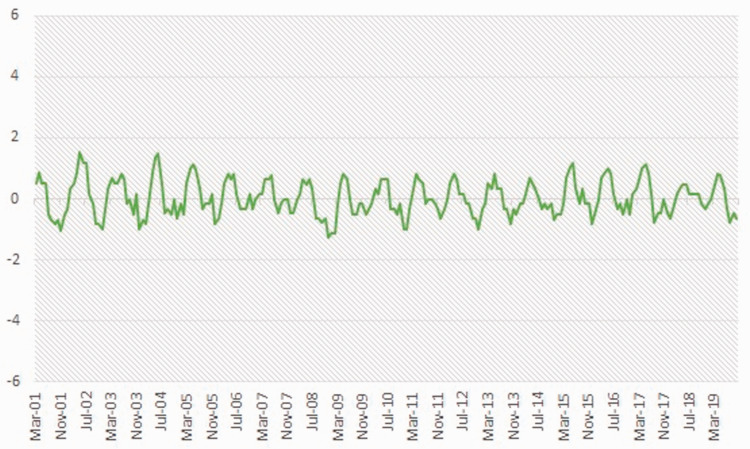
British Columbia. Per cent change in employment compared to previous month (3 month moving averages; unadjusted for seasonality for the last five months of data. Source: Statistics Canada. 2019. Table 14-10-0293-01: Labour force characteristics by economic region, three-month average, unadjusted for seasonality, last five months. Ottawa: Statistics Canada.

Below, Statistics Canada data from 2001 to 2019 are used to demonstrate the pressures that must be confronted by businesses in these volatile resource-based economies. Figure 1 depicts the percentage change in employment rates compared to the previous month using a three-month moving average for the Northeast Economic Region in BC. In this resource-based region, the fluctuations or per cent change in the employment rates vary by as much as 4–6%. By comparison, the provincial fluctuations or per cent change in employment rates vary by less than 2% (Figure 2). Rather than coping with the pressures of growth or decline, businesses in the Northeast are forced into a perpetual state of readiness to react to both. These pressures prompt questions about how local businesses in resource-based economies can mitigate the rapid boom and bust patterns associated with the turn-key operations of mobile labour.

## Supply chain vulnerabilities

Within volatile staples-dependent economies, it is critical for rural stakeholders to understand how to manage supply chain vulnerabilities in order to strategically target supply chain opportunities with existing capacity or to assess capacity gaps that need to be addressed in order to be ready and better positioned for these opportunities. In preparation for vulnerabilities across boom and bust cycles, businesses must incorporate risk management strategies to plan for different types of industry pressures (Browne et al., 2011). Industries transfer many risks to primary contractors, sub-contractors, consultants and suppliers engaged in different components of major industry resource development projects through insurance, exclusion or indemnity clauses in contracts (Lyons and Skitmore, 2004). Risks are assumed by businesses which arguably control the tasks and responsibilities, but these arrangements become more complex when considering market conditions and time constraints (Schramm et al., 2010). Construction is a particularly risky phase of resource development projects. Financial risks are exacerbated by delays, changes, or cancellation of construction projects due to fluctuations in commodity prices and exposure to poor exchange and interest rates (Almeida et al., 2017; Bandaly et al., 2013). This introduces periods when employees and equipment assets are not deployed in the post-exploration and construction season. As a result, financial risks emerge as businesses manoeuvre ways to strategically scale-up and scale-down their assets while charting opportunities for expanding their business (DPRA Canada Inc., 2013). In some cases, local businesses may have limited expertise and experience with risk analysis and management processes (Lyons and Skitmore, 2004).

The ability of rural businesses to manage supply chain vulnerabilities is further shaped by their capacity, as demonstrated through their understanding of complex procurement processes and requirements, access to flexible financial and human capital assets and infrastructure. Due to the small local economies that exist in many rural and remote regions, for example, the scale and specialization of supplies and services that are needed to support the construction and operations of large-scale industry projects do not always make their engagement in procurement processes practical (Acheampong et al., 2016; International Petroleum Industry Environmental Conservation Association (IPIECA), 2011). Businesses that pursue opportunities often struggle to work through complex registration systems (Ablo, 2017), as well as strict pre-qualification and contracting requirements that can shape the capacity and agility of local businesses to scale-up quickly and take advantage of supply chain opportunities (Esteves et al., 2013; Tordo et al., 2013). These contract requirements are especially difficult to meet during short exploration seasons in the mining sector (DPRA Canada Inc., 2013). Local businesses must register as qualified vendors with vendor management programmes after they have met pre-qualification standards, including occupational health and safety, ISO certification, management capacities and the ability to pre-finance contracts and to retain sufficient financial resources to support operational expenditures through extended payment cycles (Ahmad et al., 2016; Ramdoo, 2018). Furthermore, in volatile resource sectors, small businesses in rural and remote regions may struggle to obtain appropriate insurance coverage as local insurance brokers may be reluctant to underwrite the high capital risks associated with resource development projects (Arthur and Arthur, 2014).

The agility to scale-up quickly, however, may also be limited by the large capital investments that are required for specialized equipment during the exploration and construction phases of large-scale resource development projects (Kazzazi and Nouri, 2012), and the infrequent one-time contract opportunities, in pipeline work for example, that make it difficult to sustain local businesses (DPRA Canada Inc., 2013). The capacity to engage in these capital intensive projects is further complicated by the risks financial institutions perceive with lending to small businesses operating in volatile resource-based economies (IPIECA, 2011; Lebdioui, 2020). When confronted with high interest rates for financing (Ablo, 2017), business stakeholders simply have inadequate access to affordable financing and capital that impedes their ability ramp up quickly to take advantage of such opportunities (Arthur and Arthur, 2014).

The capacity and agility of local businesses may be further challenged by limitations with effective and timely workforce development. The pace of exploration and development may be too fast, leaving little time for small contractors and suppliers to prepare their workforces or test new innovations to be ready to scale-up (Lebdioui, 2020; Tordo et al., 2013). Local businesses may lack basic business management tools and expertise to meet administrative requirements (Hall and Matos, 2010). These human capacity challenges are exacerbated by limited access to local training that has led to a shortage of skills and formal education in rural regions (Ngoasong, 2014). Furthermore, as local businesses seek to recruit and retain a competitive workforce, they face difficulties competing with industry wages (Östensson, 2018).

Infrastructure deficits, such as with specialized equipment, power, broadband networks and computer requirements, may not well position local businesses to have the capacity to scale-up, grow and compete for supply and servicing contracts (Acheampong et al., 2016; Tordo et al., 2013). Existing equipment may have limited uses in other industries and resource sectors as businesses look for ways to respond to economic pressures and expand supply chain opportunities (Östensson, 2018). Due to inadequate information infrastructures, procurement information may not be readily available to local businesses or may reflect poor industry communication about procurement opportunities and project updates (Arthur and Arthur, 2014; Ramdoo, 2018). Despite literature detailing the challenges of scaling up and the challenges encountered during periods of decline, few researchers have explored how rural businesses ‘respond’ by deploying strategic actions to manage their assets and resources in order to maintain their viability and resilience in these volatile economic conditions (DPRA Canada Inc., 2013; Purvis et al., 2016).

## Supply chain resilience

Literature on supply chain resilience provides a useful approach to frame the fluctuating market conditions in which rural businesses in resource-dependent regions must be ready to scale-up or scale-down. The concept of resilience, however, is fraught with many definitions and interpretations of terms such as agility, flexibility, readiness, responsiveness, robustness and adaptability (Purvis et al., 2016). For our purpose, supply chain resilience is generally understood as:

the ability of a supply chain to prepare for and/or respond to disruptions, to make a timely and cost effective recovery, and therefore progress to a post-disruption state of operations but also in terms of its ability to re-emerge in ideally, a better state than prior to the disruption thereby gaining ground on the competition by bouncing back or taking advantage of new opportunities better than other firms that were affected. (Scholten et al., 2020: 1)

Under this approach, resiliency moves beyond crisis management, with businesses engaged in readiness, response and recovery (Chowdhury and Quaddus, 2016; Hohenstein et al., 2015). During the readiness phase, businesses engage in ‘proactive’ activities to predict potential threats and risks by using networks to share information, training staff to be flexible and monitor risks, strategic management of inventory assets, establishing contingency plans and establishing early warning indicators (Hohenstein et al., 2015). In small and remote communities, however, the presence of a small number of local businesses often means that there are only one or two types of a specific business that is able to thrive. Under rapidly changing market conditions, theories of networks, competition and strategy suggest that there can be challenges to share expertise and advice across ‘geographically dispersed’ businesses that have varied resources (Scholten et al., 2020). Contingency plans and redundancies through investments in extra inventories or maintaining alternative suppliers in order to enhance the capacity to respond to disruptions has increased concerns about rising operational costs (Purvis et al., 2016).

Readiness has been strengthened, however, by negotiating non-cancellable contracts and forward contracts^1^ to manage these financial risks (Almeida et al., 2017). Others have pursued flexible contractual arrangements with pricing, deliverables and delivery dates (Bandaly et al., 2013). Local businesses have improved their agility^2^ and resiliency across economic cycles by enhancing their understanding of industry standards and market conditions, as well as by diversifying the knowledge and skills of their staff to be ready for new opportunities (DPRA Canada Inc., 2013; Yusuf et al., 2014). Readiness is not a one-time investment, however, but reflects an ongoing renewal of assets and long-term strategies to best position businesses to be flexible and responsive to rapidly changing economic conditions. For businesses, part of the challenge associated with readiness is attributed to the transformative scale of economic change and the capacity of local stakeholders to cope with the increasing pace of transition (Markey et al., 2012).

As turbulent events unfold, ‘reactive’ responses are then mobilized to stabilize operations. The resilience exhibited during this phase depends on the capacity of businesses to sense a disruption event in order to minimize the time needed to mobile appropriate responses (Purvis et al., 2016). The recovery phase represents a series of actions to return the business to its original state of operations. Businesses may then strategically use learning and innovation to support growth and improve competitive positions (Hohenstein et al., 2015). Across these stages of resilience, the implementation of a risk management culture is important to not only anticipate, identify and monitor risks, but also to mobilize more efficient recovery strategies (Chowdhury and Quaddus, 2016).

Researchers argue, however, that we know little about supply chain resilience beyond ‘top-level generic supply chain strategies’ with limited attention to situate supply chain management practices in the wider context in which firms operate (Scholten et al., 2020: 2). Our research focused on the engagement and strategic actions of businesses in supply chains related to mobile workforces as the lens or motive to expand the literature about how these supply chains are embedded in broader pressures unfolding through boom and bust resource-based economies in rural and remote regions.

## Context

**Figure 3.**
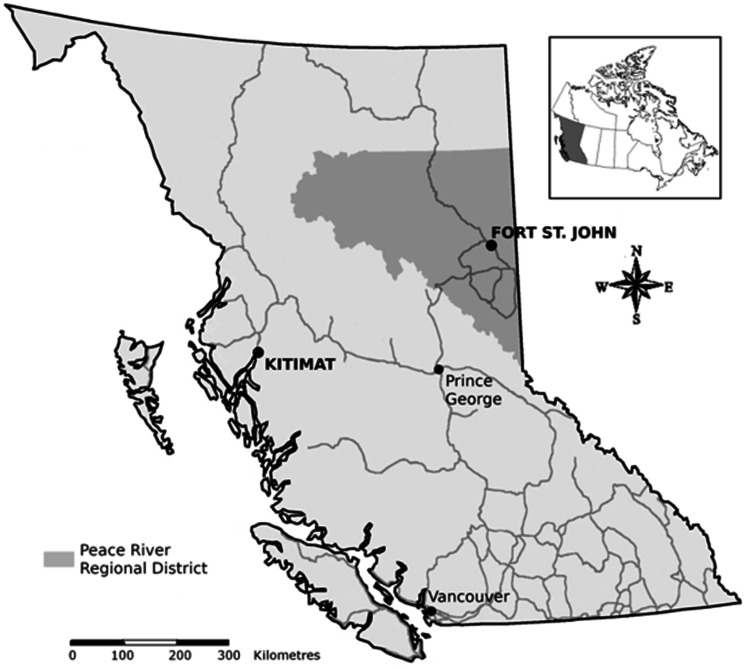
Study site.

Building upon previous research that focuses on supply chain opportunities associated directly with large-scale industrial project components, this research examines how businesses in Fort St. John (Figure 3) scaled-up and scaled-down to capture benefits from the mobile workforces associated with projects in the Northeast region of BC through local content and supply chain processes. Fort St. John is a municipality consisting of 20,155 residents, a population increase of almost 51% over 30 years (Statistics Canada, 2016). The city is surrounded by rural, unincorporated areas that are served by the Peace River Regional District.

**Figure 4.**
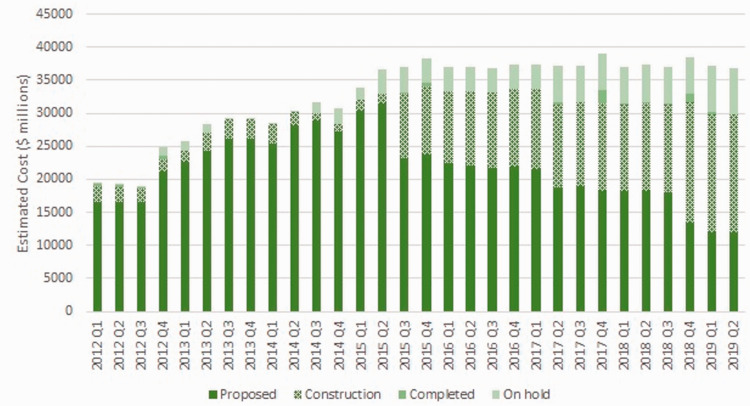
Estimated costs of Northeast Major Projects. Source: Ministry of Advanced Education, Skills and Training. 2012–2019. British Columbia Major Projects Inventory. Victoria, BC: Ministry of Advanced Education, Skills and Training, Province of British Columbia.

The Northeast Economic Region is predominantly driven by oil and gas, mining and forestry. With almost $37 billion in estimated costs between 2012 and 2019, there are many large-scale projects unfolding in the Northeast Region of BC that provide opportunities for mobile workers (see Figure 4). Two key projects unfolding in the region include BC Hydro’s Site C Project^3^ and the Coastal Gaslink natural gas pipeline project.^4^ Construction has also been in progress for methanol plants, biomass plants, wind energy projects, coal mines, transmission line projects, natural gas processing plants and other pipeline projects. Construction on these projects has accelerated since 2015; although, project delays and changes to commodity prices has meant that a number of these projects have since been put on hold (Ross, 2018).

Fort St. John does not have any work camps operating within its municipal boundaries; however, there are many work camps nearby in the surrounding rural areas that accommodate workers engaged in hydro, oil and gas (drilling rigs, compressor stations, pipeline construction), mining, rail and forestry activities (production facility maintenance, forest fire fighting, reforestation). This includes the up to 1700 person Two Rivers Lodging Camp that provides lodging for mobile workers at BC Hydro’s Site C Project and is located adjacent to the municipality. Mobile workers are also extensively accommodated in hotels, campgrounds and rental properties throughout the community.

## Methodology

Using an in-depth qualitative approach, 45 key informant interviews were conducted with businesses located in Fort St. John. Business stakeholders were selected for their potential to provide information that can help to better understand the issues that are shaping challenges and strategic actions associated with scaling up and scaling down to capture benefits from mobile workforces. Business opportunities transcend construction, operations, decommissioning and re-opening of camp facilities. Businesses varied in size with workforces between 10 and 80 employees, and were engaged in food, beverage and catering; rental services; hotels; construction; retail and personal services; communications; waste management; safety; and business services related to mobile workforces (Table 1). Business opportunities for the companies were pursued across a range of industry stakeholders and sectors, including with AFDE,^5^ Alta Gas, ATCO, CN Rail, CRNL/Septimus, Petronas, Peace River Hydro Partners (PRHP), drilling rigs, remote compressor stations, general mobile camps, forest fire camps, reforestation camps and camps that support the shutdown servicing of the oriented strand board plant.

**Table 1.** Local business opportunities related to mobile workers and camps.

Accommodations and food	▪ Camps, crew houses, hotels/motels▪ Provision of catering/catering equipment, alcoholic/non-alcoholic beverages, grocery supplies▪ Provision of water, portable fresh water supply
Construction	▪ Setting camps and camp offices up, drywall, roofing, siding/weather proofing, coordinating set up with ramps and trailers, receiving modular units, concrete work, building access and sidewalk curbs, barriers/lock blocks, installing windows, interior finishes▪ Setting up super-temporary/extended camp units ▪ Equipment: generators, lighting, equipment and vehicle rentals
Maintenance	▪ Maintenance/camp repairs▪ Waste management; waste and recycling bins; domestic waste; storage tanks; dust control▪ Carpet cleaning for camps/crew houses▪ Laundry services
Communications	▪ Wiring communications▪ Wi-Fi▪ Telephone/cell phone service
Safety and Security	▪ Inspections (operations/re-opening of camps), fire extinguishers▪ Locksmithing for rentals/camps▪ Safety training/certification, drug and alcohol testing
Office	▪ Printing: business cards, safety permits, laundry tickets, etc. ▪ Provision of printers, scanners and other office equipment▪ Meeting space for upper management meetings
Retail and Recreation	▪ Retail/salon services▪ Events/tournaments for mobile workers▪ Recreational amenities
Decommissioning	▪ Reclamation, hauling away debris / waste

Participants were asked about actions needed to get ready for the influx of mobile workers, such as addressing contract requirements, investments, or training and recruitment; events or sudden changes in industry strategies that may have affected their business with work camps or mobile workforces (i.e. project delays, commodity prices); and challenges to mobilizing strategies to scale-up or scale-down in response to fluctuations with mobile workforces and match the speed of industry changes.

Following each interview, summary notes were provided to participants for review. Once a final summary file was completed, latent and manifest content analysis was done to identify, code and categorize patterns and themes that emerged from open-ended questions (Neuendorf, 2016). To improve reliability, members of the research team worked together to code and categorize themes in order to develop a common coding approach. As new themes emerged, they were evaluated across the team during the course of multiple rounds of coding. To complete the manifest content analysis, the research team consolidated information about business engagement with mobile workforces based on a range of topic areas. By highlighting key words, researchers were able to create a series of categories and sub-categories (Andersen and Svensson, 2012). Through latent content analysis, deeper meanings and connections across the themes were explored.

Due to the in-depth, exploratory nature of these interviews, our intention was to provide a foundation for further investigations and the development of supportive policies and programmes that strengthen the agility and resilience of businesses seeking to maximize benefits from mobile workforces. Our findings, as such, must be considered with some study limitations. This is not a longitudinal study tracking business decisions and strategies across economic cycles, but rather a retrospective approach to explore how business leaders prepare for, and respond to, supply chain pressures. A key limitation with this approach is the ability of participants to accurately and willingly recollect and distinguish details (Barnes, 2001) across chaotic and competitive conditions associated with the regional waves of different resource-based sectors. The scale of the study and our long-term research relationship with the region does, however, provide a robust portrait of business strategies and actions engaged in these volatile supply chains.

## Findings

In Fort St. John, supply chain opportunities with mobile workforces and camps have been impacted by industry changes such as commodity price fluctuations, economic downturns, delays with liquefied natural gas pipeline projects (i.e. Trans Mountain and Coastal Gaslink), curtailments in the forest sector and cancellation of work (Financial Post, 2019; Lough, 2018); although, there has been rapid growth associated with the exploration and construction of large-scale industrial projects. Opportunities to serve mobile workforces engaged in BC Hydro’s Site C project, for example, helped to level the impacts of downturns in other sectors. To prepare for, and respond to, rapid changes in resource-based economies, businesses adopted a series of strategies to bolster their agility, capacity and readiness for change. Local businesses, however, confront many challenges as they scale-up and scale-down in response to fluctuations with mobile workforces and industry activity in the region. To explore how businesses manoeuvred rapid changes to supply chains associated with large mobile workforces, our findings explore the challenges and responses in relation to eight key themes that emerged from the data, including communication, access to capital, insurance, land development, infrastructure, managing inventories, human resources and diversification.

### Communication

During the tenure of supply and service contracts, readiness to scale-up and scale-down has been complicated by critical communication issues with camps. There may be limited notice given for when camps are opening, closing, or re-opening in order to facilitate timely strategies to scale-up. As one local business stakeholder told us:

There isn’t usually a lot of notice. I think the one big camp that we had was a couple of weeks’ notice before they resized and closed down. Then we had one shut down for a couple months and they just started up again. It was just like oh we have an email from them. I guess they are up and running. There is not a lot of communication. It’s just whether we get an email or not for orders . . . (Participant #11, 2019)

Many camps do not have permanent or pop-up offices in Fort St. John; instead, many camp head offices are located in the neighbouring province of Alberta. This makes it difficult for local businesses to find information about camp contacts, a challenge that is exacerbated by frequent turnover of camp contacts. It means that there is a lack of information about supply chain opportunities related to mobile workforces and work camps in order to better position local suppliers and contractors to be ready to mobilize their assets in advance of camp development.

Early warning signs between camp operators and their contractors or suppliers may be limited or non-existent. Limited to no notice of camp closures or cancellation of hotel bookings exacerbates the risks for local businesses that decline other business opportunities due to arrangements with industry to accommodate mobile workforces. In some cases, business plans were based on population projections that included mobile workforces. Collectively, these communication issues directly impact the ability of local businesses to develop, mobilize, or redeploy their assets as needed in these supply chains.

### Access to capital

Access to capital was a key issue as businesses worked to scale-up their operations in advance of the influx of large mobile workforces. Business owners relied upon family shares, business loans from Indigenous organizations, the Business Development Bank of Canada, or drew from their own accumulated capital. There were businesses, however, that had limited access to equity. New or younger entrepreneurs with more limited levels of equity in their home or bank assets struggled to obtain good financing arrangements, and were required to provide higher down payments for commercial buildings.

Capital pressures were exacerbated by financial institutions that do not always understand resource-based economies. Some participants felt that financial stakeholders in larger urban centres may not listen to local staff to understand the needs of businesses and the resource sector, often resulting in decisions to not approve loans for businesses in rural and remote regions. With a focus on real estate, there were also some participants who thought financial lenders may not understand the ability to move large vehicles and pieces of equipment to other locations where it can be sold during economic fluctuations. Businesses that shed assets during the downturn are then challenged to buy those assets back as they ramp up for renewed economic development. For example, a stakeholder explained:

So when people don’t live here and make decisions financially for some of the people, that’s another impact for those that are trying to be nimble and be able to take advantage of opportunities quickly. Financial institutions that are in the Lower Mainland look at a credit application for a Kenworth truck and they don’t understand it. If they can’t secure it by a condo in Burnaby, then it’s too high risk and they will rate it and not approve the credit . . . We have a whole generation of lenders that don’t understand anything except real estate. They cannot get their brain around the fact that you can take that million dollar Kenworth and you can actually physically move it to somewhere where it will sell. (Participant #20, 2019)

These decisions impact the ability of businesses to be nimble and take advantage of opportunities quickly.

New entrepreneurs entering supply chains can get caught in boom and bust cycles when pressures surrounding investments, mortgages and lease arrangements impede their flexibility and capacity to scale-down. Inappropriate management of capital impacts the engagement of local businesses in supply chains as businesses either close due to under capitalization or due to over spending. By comparison, better capitalized companies are positioned to absorb infrastructure and capacity before scaling up during the next period of rapid development.

### Insurance

Business stakeholders found that insurance companies do not always understand the oil and gas sector and the suppliers and contractors that serve them. Bonding companies did not always want to assume the risks associated with resource sector activities. Since the insurance sector is not confined or restricted to provincial boundaries, some businesses have reached out to insurance companies in the province of Alberta to engage in these conversations. Despite these actions, businesses find it difficult to obtain the various levels of required insurance for different companies as a part of their supplier requirements. The result is that some may have their insurance rejected as they attempt to engage in supply chain opportunities. Some struggle with changes to insurance requirements and insurance packages from year to year that can affect their response to economic events. Furthermore, agricultural suppliers struggle to obtain insurance for small acreage crops or to cover delays or changes with camp supply orders.

### Land development

Limited space to scale-up and scale-down operations can be an issue as demand for vehicles, equipment and storage space for food can vary significantly. Local businesses may need access to a lot of property for a short period of time. As one grocery chain stakeholder noted:

Space is becoming an issue. Especially because our online shopping has now taken over our camp area, so it’s a struggle where we are going to put the camp workers. Luckily, it’s only usually two days that we have to deal with it and then they are gone, but there’s a lot of boxes because we have to keep stuff in coolers. We have to keep stuff in the freezers, and we have to keep stuff dry. We always need more space. Our warehouse is not big enough. (Participant #11, 2019)

Similarly, a vehicle rental company representative explained:

For me, it’s just the space just because my fleet size varies so drastically. Right now, my lot out back is almost empty and we have got 5 acres here because everything is out on rent. You take two months ago when break up started, it was really hard to find a parking spot back on 5 acres. I had almost 200 trucks here. I mean trucks aren’t small, so it’s not like your trying to park a widget or a screw or a bolt. That’s the property constraint. I need a lot of land for a short period of time. (Participant #31, 2019)

As businesses work to scale-up quickly, prolonged land development processes through local government were deemed to increase costs, and therefore affected the ability of local businesses to compete in supply chains. The concern is not only with the length of time to review development plans, but also challenges to receive a thorough review on the first submission. Businesses may receive requests by local government to revise one component of the plan, but may be required to make new revisions under a second review of revised plans. As one participant argued:

Yeah. Sending your plans in and getting them reviewed and then they will send it back and say you have to change this on page 4 on your plans and then you send it back and then they say oh you need this on page 6. Rather than read the whole thing and say you have to do this, this, and this . . . They let you get invested and they tell you a little bit of stuff at a time and now you can’t back out. They were going to build 3 buildings in one location and the City created so many complications for the first one. That’s where it sits. They built the first one and I don’t think they’ll build the other two. (Participant #3, 2019)

Complex senior government consultation processes to access or lease new land can increase development costs. As businesses move to ramp up quickly, some struggle to determine all the senior government agencies that must be contacted. Businesses must complete a 30-day consultation period with stakeholder groups (i.e. First Nations, landowners, etc.), but these consultation periods are often extended, and, in some cases, have taken up to a year to complete. Not only have costs increased due to such delays, but the risks for local businesses have amplified as clients have threatened to walk away.

### Infrastructure

To be ready to pursue opportunities associated with mobile workforces, business infrastructure investments focused on equipment and vehicles to support the mobility of workers; to support construction and servicing arrangements with camps; and to support recreational opportunities (Table 2). Physical infrastructure investments in building assets focused on renovating hotel and commercial space to accommodate mobile workforces and to manage supplies and equipment assets. To meet contract requirements, there were also investments in communications infrastructure in order to provide high speed Internet in remote areas. There were infrastructure gaps, however, to address underdeveloped supply chain opportunities for agricultural producers. A washing facility is needed to enable agricultural producers to prepare clean bulk vegetables for camps that are currently transported in from other regions.

**Table 2.** Investments to ramp up.

Equipment and vehicles	• Trucks, vehicle equipment (i.e. flags, radios, appropriate tires, first aid kits), shuttle vehicles, rental equipment, cleaning equipment, testing equipment, drug and alcohol testing equipment, portable equipment to program / chip keys, catering equipment and new power golf carts
Physical infrastructure	• Renovations to modernize hotels / building spaces; resurfaced parking lots; and investments in shop space, storage space for products and checkout space for camps
Communications infrastructure	• Tower sites, higher quality microwave infrastructure and related equipment for high speed Internet in remote areas

As businesses scale-up during periods of rapid growth, past experiences and lessons from losses incurred over boom and bust periods have led to cautious infrastructure investments. Some businesses were less likely to commit to expensive monthly lease arrangements during early stages of growth as the economy has periodically deflated. Rental fleets can also fluctuate over boom and bust periods, prompting some to be cautious about purchasing or financing equipment that will require long-term payments. Some food and equipment suppliers prefer to purchase used equipment to avoid significant business debts and mitigate risks and vulnerability. With limited management experience over boom and bust cycles, however, new entrepreneurs may scale-up too quickly when investing in new, used, or leased equipment. As businesses scale-down, some are reducing the building space that is owned or rented in order to avoid overhead costs for space that is no longer used. Project delays and economic downturns have led to the sale of equipment and adjustments to diversify or reduce risks with stock inventory.

### Managing inventories

As businesses work to improve their resiliency in supply chains, scaling up and scaling down strategies must consider the implications for product and equipment inventories. During economic upswings, camps and mobile workers often need products and supplies (i.e. clothing, protective gear and equipment) that can take weeks to order and receive due to the remoteness of the community. It is difficult for small businesses to maintain stock in large quantities due to the uncertainty associated with boom and bust cycles, and due to manufacturing requirements for minimum orders that can pose substantial risks for businesses in volatile resource-dependent economies. Local businesses have struggled to plan inventory investments with manufacturers. Delivery for vehicles and larger pieces of equipment, for example, can require extensive timeframes. To be ready, some businesses assume the risk of maintaining larger inventories or draw from nearby inventories with affiliated business locations in other regions. As one stakeholder explained:

They’ll know they got the contract, but until they have the signed agreement back, they don’t order anything from me. XXXX knew they were awarded the contract in last May. Last August long weekend, they didn’t have a signed agreement, so they couldn’t pull the trigger with any of their vendors. I was expected to have 100 trucks which is 4 million dollars. It’s hard to plan for and then of course they finally get it and it’s oh can you start Monday. The tough part is planning with the vehicle manufacturers for me. At best, you have a two-month delivery time from the time you order a new truck to having it delivered here. Earlier this year, for example, Ford was twenty weeks to wait to get one F350 truck. I can’t sit on that and rely on waiting for it to come in. So we keep a lot of inventory in stock and we carry extra stock just for those things. (Participant #31, 2019)

Agricultural suppliers face a unique set of challenges to manage inventories as they work to scale-up and engage with mobile workforces. Seasonal crops may already be allocated to buyers by the time a camp reaches out to producers. For general retail, uncertainty with camp orders during periods of economic decline can result in product bottlenecks and storage space issues. Inventories may also be impacted by camp requests to purchase more generic brands in lieu of brand name products to reduce costs.

### Human resources

Businesses are concerned about job losses during economic downturns as they scale-down their operations, but their ability to regain those human resources as they recover is equally challenging under competitive labour market conditions. As industry activity increases, local businesses are caught between clients that expect low rates, and labour expectations for higher wages as the economy recovers. Businesses struggle to reconcile these recruitment and retention pressures that reduce margins. As this stakeholder noted:

I knew a downturn would be tough; but what I didn’t see was a bit of an upturn being tougher because the industry when it’s down . . . people expect it to be down, but as soon as things ramp up, businesses get caught in between our clients expecting extremely low rates and people noticing that things are getting busier and demanding higher wages. So the margins are already tight and people want more. (Participant #19, 2019)

Even as companies scale-up, some have been reluctant to commit to long-term employees due to the uncertainty associated with pipeline projects and the scale of mobile workers that will be needed to support those projects. In some cases, businesses use short-term contracts (i.e. 3–6 months) during the early growth phase of resource development cycles. Others have adjusted their human resource strategies to avoid excessive hiring as they scale-up in order to avoid layoffs and lost capacity during economic downturns. Strategies have also been deployed to restructure staff roles, reassign front end staff, or draw from affiliated regional offices in lieu of new hires. Under these volatile conditions, the ability to ramp up quickly is also impacted by the training required, prompting businesses to ensure they have an efficient and complete orientation process in place.

### Diversification

Finally, as a further response and recovery strategy, businesses diversified their operations beyond the oil and gas sector with opportunities through BC Hydro, forestry and forest fire fighting camps, mining and construction to reduce business risks and remain competitive. Risks were offset by pursuing corporate contracts that have regular fixed timeframes. For example, the provincial government’s contracts to service forest fire crews, which are not exposed to the vagaries of boom-bust cycles, provide predictable opportunities that support and stabilize business plans.

Products were diversified by expanding into organic food production, retail products for female mobile workers, asbestos removal and new communication technologies, as well as modifying existing equipment to meet varied rental equipment needs. Services have been streamlined to focus on those that are most relevant to a mobile workforce (i.e. from strength training to drug and alcohol testing).

## Discussion

In volatile, staples-dependent, economies there are significant opportunities for local businesses to capture more local economic benefits through increased participation in the supply chains serving mobile workforces and work camps. This supports the more general desire by rural regions to derive a greater share of the benefits from large-scale resource extraction. We focused on local business participation in mobile workforce/work camp supply chains because much of the current literature is focused only on the resource extraction activities themselves (Ablo, 2017; Esteves et al., 2013; Tordo et al., 2013). In studying opportunities for local businesses we found successful strategies and significant constraints relative to the readiness, response and recovery by those businesses as they engage in highly fluctuating boom and bust economic cycles.

Our contributions to broader theoretical and practice-based debates are two-fold. First, our research extends the literature around local businesses from engagement with the exploration, construction and operations of resource-based extraction to include engagement in the supply chains supporting the large mobile workforces of those extraction activities. Local businesses not only engage in the construction, maintenance and decommissioning of work camps, but also support their operations through the provision of food, security, waste management, communications, material and office supplies, general servicing and recreation. While our findings describe the breadth of these supply chains, there is a lack of information about their ‘value’, and the extent to which there is economic leakage to suppliers and contractors outside of the region. These gaps limit the advance of policy and practice around local content benefits for rural and remote regions and require further investigation (Tonts et al., 2013).

Second, our inquiry expands the understanding of the risks, capacities and strategies deployed by small local sub-contractors and suppliers. Guided by the unique constraints of rural and remote regions and the themes that emerged from this research, we have strengthened the framework to understand how local businesses pursue readiness, response, recovery and growth against risky and highly fluctuating economic conditions (Ryser et al., 2019; Haslam McKenzie and Rowley, 2013). The capacity to scale-up and scale-down in response to changing conditions is shaped by human resource and infrastructure strategies, careful inventory management and well-organized contract management strategies. Large inventory redundancies are used to counter challenges ordering and receiving stock in rural and remote regions. Furthermore, long-term contracts are often based on expectations of stability or continued growth (Purvis et al., 2016) that are not realistic in volatile staples-dependent economies. As a result, local suppliers have also adapted by seeking stable, predictable short-term government contracts as backstops. Businesses have also pursued ways to improve their agility by engaging in mobile workforce supply chain opportunities across different resource sectors.

The readiness of local businesses is constrained, however, by limited access to information about supply chain opportunities related to mobile workforces – a problem compounded by limited networks with camp operators (Hohenstein et al., 2015; Scholten et al., 2020). Too often, by the time local work camp opportunities are identified, camp operators already have external supply chains in place. It can be difficult for local businesses to break into, and compete with, well established external supply chain networks. Urban-centred capital and insurance brokers further weaken the readiness and agility of these businesses to plan and recover through their inadequate understanding of these economic sectors. Moreover, our research expands upon previous work by demonstrating how human resource management challenges transcend bust and recovery periods as labour expectations test a recovering business sector capacity and rural contractors and suppliers struggle to compete with large-scale industry wages. As such, investments in highly skilled or lean teams (Purvis et al., 2016) are not always possible in rural and remote regions where small labour markets are highly competitive, and where there are limited education and training options to develop and renew human capital.

State policies and programmes have a number of roles to play in supporting local businesses as they become more adept at scaling up and scaling down servicing for mobile workforces (Lebdioui, 2020). Provincial and federal government review, consultation and project approval processes can better assess how their policies and regulations (or lack of) impact the ability of local sectors to engage in supply chains to serve mobile workforces. To support the readiness and responsiveness of these businesses, early and clear access to information around mobile workforce/work camp supply chain opportunities is needed. In this regard, provincial governments should designate a lead government body to develop and maintain a central registry of mobile work camps (with current information about the location, size and contact information for the camp procurement team) as well as a project registry (with similar information) on proposed mobile work camps. Provincial and federal policies and processes can strengthen the readiness of local businesses by developing and enforcing requirements for industries and work camps to promote supply chain opportunities at the local level.

As local businesses seek improved access to capital to improve their agility, we suggest that provincial and federal capital programmes need to be restructured to better position businesses to manage the opportunities and vulnerabilities associated with mobile workforce/work camp supply chain opportunities (Arthur and Arthur, 2014; Lebdioui, 2020). Moving forward, more research is needed to better understand how resource-based economies are informing access to capital policies amongst financial and government institutions. To what extent do capital deficiencies stemming from institutional policies, for example, weaken local supply chain networks and limit benefits accrued to local economies? These pathways of inquiry should also examine the roles of senior governments to address infrastructure deficiencies that also limit the agility of local businesses to maximize benefits from mobile workforce supply chains. As resource-based communities continue to experience volatility over boom and bust cycles, local governments can support the agility and resilience of businesses by facilitating dialogue between industry, work camp and local businesses. Local governments also have a role to play in local land use designations and permitting to better enable local business flexibility. Questions remain, however, about how local government policies are being renewed to reflect this new landscape. Competitive property tax rates and streamlined development permit (and related) processes, for example, can affect the agility of businesses to scale-up quickly when new opportunities emerge.

## Conclusion

In rural and remote staples-dependent places and regions, past business approaches were designed to reflect labour that was rooted in place and industry that acted in a paternalistic relationship with the community. These circumstances have changed dramatically and there is a need to update business practices, and to seize business opportunities, linked to an increasingly mobile labour and capital landscape. Strengthening the capacity and opportunities of local businesses to service this ‘mobile’ sector can help to diversify the economy, reduce economic leakage and expand opportunities for those businesses beyond existing upstream industry activities. These findings provide insights to guide more prepared and flexible business engagement in rural and remote supply chains addressing the needs of mobile workforces. Moving forward, a broader investigation of these issues is needed to understand the scale and scope of impacts that mobile workforces have on local businesses, and to distinguish which supply chain issues transcend different market conditions and boom and bust periods as industries respond to market fluctuations.

## Notes

1 A forward contract is an arrangement to buy or sell goods and services at a future date.

2 Agility refers to the competitive attributes of a business through their capacity to be flexible, proactive, innovative and respond quickly to changes in the business environment (Yusuf et al., 2014).

3 BC Hydro’s Site C Clean Energy Project proposes to develop a third dam and hydroelectric generating station on the Peace River, providing up to 1100 MW of capacity and 5100 GWh of energy annually. The estimated cost of the project is $10.7 billion (Cdn). Construction began in 2015.

4 The Coastal Gaslink natural gas pipeline project will be roughly 670 km in length to transport natural gas from the Peace River region in northeast BC to a facility in Kitimat. The estimated cost of the project is $6.2 billion (Cdn).

5 AFDE is a partnership of four construction companies engaged in the Site C Project for BC Hydro, including Aecon, Flatiron, Dragados and EBC.
